# DNApi: A *De Novo* Adapter Prediction Algorithm for Small RNA Sequencing Data

**DOI:** 10.1371/journal.pone.0164228

**Published:** 2016-10-13

**Authors:** Junko Tsuji, Zhiping Weng

**Affiliations:** Program in Bioinformatics and Integrative Biology, University of Massachusetts Medical School, Worcester, Massachusetts, United States of America; CNRS UMR7622 & University Paris 6 Pierre-et-Marie-Curie, FRANCE

## Abstract

With the rapid accumulation of publicly available small RNA sequencing datasets, third-party meta-analysis across many datasets is becoming increasingly powerful. Although removing the 3´ adapter is an essential step for small RNA sequencing analysis, the adapter sequence information is not always available in the metadata. The information can be also erroneous even when it is available. In this study, we developed DNApi, a lightweight Python software package that predicts the 3´ adapter sequence *de novo* and provides the user with cleansed small RNA sequences ready for down stream analysis. Tested on 539 publicly available small RNA libraries accompanied with 3´ adapter sequences in their metadata, DNApi shows near-perfect accuracy (98.5%) with fast runtime (~2.85 seconds per library) and efficient memory usage (~43 MB on average). In addition to 3´ adapter prediction, it is also important to classify whether the input small RNA libraries were already processed, i.e. the 3´ adapters were removed. DNApi perfectly judged that given another batch of datasets, 192 publicly available processed libraries were “ready-to-map” small RNA sequence. DNApi is compatible with Python 2 and 3, and is available at https://github.com/jnktsj/DNApi. The 731 small RNA libraries used for DNApi evaluation were from human tissues and were carefully and manually collected. This study also provides readers with the curated datasets that can be integrated into their studies.

## Introduction

Small RNA sequencing profiles the abundance of small RNAs (typically shorter than 35 nucleotides) such as small interfering RNAs, microRNAs, and PIWI-interacting RNAs [1]. The number of small RNA sequencing datasets in public repositories has been increasing rapidly [2], and meta-analysis of multiple datasets can provide novel insights, for example, [3] and [4].

Adapter removal is an essential step in computational preprocessing of small RNA sequencing libraries, because only those reads that contain the 3´ adapter are full-length small RNAs and kept for downstream analysis [5]. Many software packages can perform adapter removal with a given 3´ adapter sequence [6–9]. However, the adapter information is often not available and can be erroneous in the metadata. Furthermore, the 3´ adapters of multiplexed small RNA libraries contain barcode sequences, which makes preprocessing more challenging. As of November 2015, the Gene Expression Omnibus (GEO) repository contained 1,708 small RNA libraries from human tissues sequenced using the Illumina platform, but only 539 of them provided information on adapter sequences either in the GEO entries or in the supplemental materials of the publications cited in the metadata. Roughly half of these libraries (267/539) were de-multiplexed libraries and had barcoded 3´ adapters.

We have developed DNApi, a de novo adapter prediction (iterative) algorithm for small RNA sequencing data. Using the 539 publicly available small RNA libraries accompanied with the 3´ adapter sequences, we evaluated the performance of DNApi and found that DNApi can accurately predict 3´ adapter sequences including barcoded 3´ adapters from the reads directly. Furthermore, in a large-scale meta-analysis, it is also important to judge automatically whether the input small RNA libraries were already processed, i.e. the 3´ adapters were removed. Given another batch of 192 publicly available processed libraries, DNApi perfectly grouped those libraries as “ready-to-map” small RNA libraries by incorporating a user-defined read-mapping tool. Through the run, DNApi also assesses the quality of small RNA libraries, which is important for meta-analysis across many libraries.

## Materials and Methods

### Small RNA datasets

For the evaluation of DNApi, we collected two types of publicly available datasets from GEO: 539 small RNA libraries in the GEO with 3´ adapter sequences in their metadata (S1 Table), and 192 small RNA libraries which were already processed according to their metadata (S2 Table). For 126 of the 539 small RNA libraries, the adapter information was not provided in their GEO metadata, but instead was contained in the supplemental materials of their publications. For these libraries, we manually extracted the 3´ adapter sequences from the publications. All the datasets were from human tissues, and the human reference genome (hg38) downloaded from UCSC Genome Database [10] was used for the read mapping step in the *exhaustive* mode (see “Implementation” for more detail).

### Quality trimming

To see how reads trimmed by the sequencing quality scores (hereafter quality trimmed reads) affect adapter prediction performance, we performed the Mott trimming algorithm used in BWA [11] and cutadapt [6]. Briefly, the trimming algorithm starts from the 3´ end of each read, subtracts a preset cutoff quality score from the quality score at each position and adds the remainder to a cumulative score at the position. The 3´ portion of the read at the position with the minimum cumulative score is trimmed off. We tested two quality score cutoffs, 10 and 20.

### Implementation

#### Adapter prediction algorithm: *single* mode

**Fig 1:**
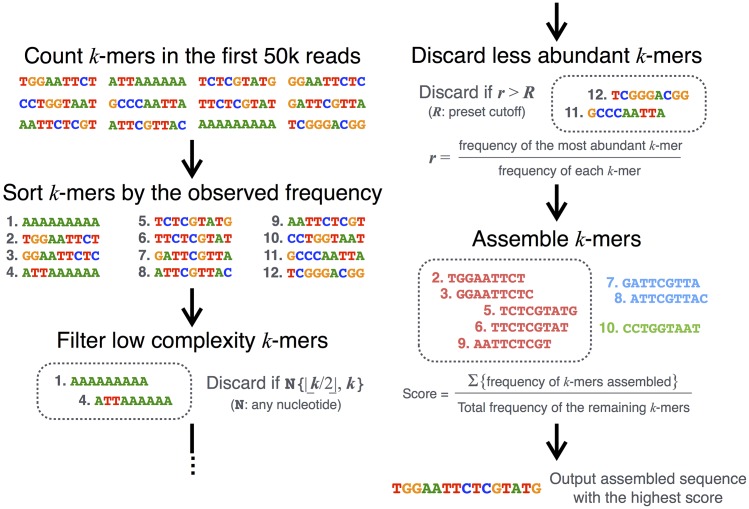
Adapter prediction algorithm workflow.

DNApi predicts 3´ adapter sequences *de novo* (Fig 1). Based on the concept that most reads are expected to contain the 3´ adapter or a prefix of it, the algorithm counts all *k*-mers in the first 50,000 reads and sorts them by frequency. To remove low complexity *k*-mers, e.g. poly(A), the algorithm discards *k*-mers containing a substring of homopolymer in length from |*k/2|* to *k*. For each remaining *k*-mer *s*, the algorithm computes the ratio, *r* = *f(S)* / *f(s)*, where *f(s)* is the frequency of *s* and *f(S)* is the frequency of the most abundant *k*-mer *S*, and then discards infrequent *k*-mers with *r* larger than a preset ratio *R*. Finally the algorithm assembles the remaining *k*-mers by suffix-prefix perfect matches. This results in multiple assembled sequences. Each assembled sequence is assigned a score, calculated as follows. We first summed the frequencies of the *k*-mers used for the assembly and then we devided the summed frequency by the total frequency of all *k*-mers in the run. The division by the total frequency of all *k*-mers in the run is for normalizing the scores across multiple runs in the *iterative* mode (see “Iterative adapter prediction: *iterative* mode”). The assembled sequence with the highest score is the putative adapter sequence. Hereafter we call a single run of this algorithm as the *single* mode.

#### Iterative adapter prediction: *iterative* mode

Our adapter prediction algorithm ranks assembled sequences to choose a sequence with the highest score as the putative adapter sequence. Although a specific combination of *k* and *R* (*k* = 9 and *R* = 1.4) can predict the correct 3´ adapter sequences in the majority of the libraries (see “Performance on adapter prediction” in Results and Discussion), different combinations of *k* and *R* are able to predict the correct adapter sequences that were missed in the setting of *k* = 9 and *R* = 1.4. We observed that the correct adapter sequence was not always ranked as the number one putative adapter, yet was nearly always among the putative adapters. This observation indicates that a tallied score of an identical assembled sequence from various combinations of *k* and *R* may improve the prediction performance.

In the *iterative* mode, DNApi runs the algorithm (Fig 1) with several combinations of *k* and *R*, reorders the assembled sequences by tallying the normalized scores in separate runs, and selects a sequence with the highest score. For the computation, we used *k* ∈ {9, 11} and *R* ∈ {1.2, 1.3, 1.4}.

#### Exhaustive adapter prediction and quality control: *exhaustive* mode

If a reference genome is available, small RNA reads should map to the reference genome with a high mapping rate after the correct adapter sequence is removed. It is also important to detect whether an input library is already processed, and this can be also judged by observing the mapping rate. In the *exhaustive* mode, DNApi runs the *single* mode with several combinations of *k* and *R*, and searches for the optimal 3´ adapter by incorporating a read mapping step with a user-defined read-mapping tool. We chose the same combinations of *k* and *R* used in the *iterative* mode in this study. For each predicted 3´ adapter, the program clips the adapter from input reads by searching the exact 7-mer prefixes of the adapter. After the adapter removal, DNApi runs a user-defined read-mapping tool with a given mapping command to map the processed reads to a reference genome. In this study, we used Bowtie [12] for mapping reads to a reference genome and required reads to map perfectly, i.e. without mismatch. DNApi deems the 3´ adapter with the highest genome-mapping rate as the optimal adapter. If the mapping rate with the optimal 3´ adapter is lower than 20%, DNApi warns the user that the quality of the input library is poor (see Results and Discussion). In addition to the adapter prediction and the quality analysis, the *exhaustive* mode provides cleansed reads for users.

## Results and Discussion

### Performance on adapter prediction

We evaluated our algorithm using the 539 human small RNA libraries in the GEO with 3´ adapter sequences (S1 Table). S1 Fig shows the sequencing quality and read length by year, indicating an increase in quality over time.

**Fig 2:**
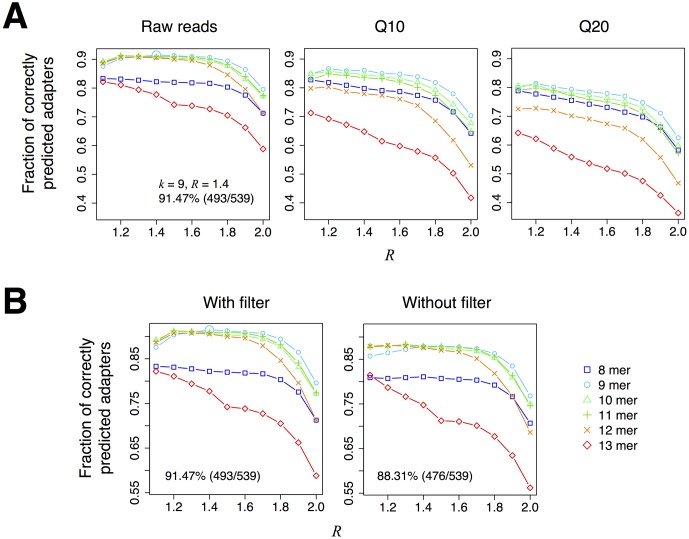
Adapter prediction performance at different parameter settings on the 539 small RNA libraries. **A**: Adapter prediction performance on raw reads (left) and trimmed reads with Phred score cutoff ≥ 10 (Q10; center) and ≥ 20 (Q20; right) is shown at different *k* and *R* parameter settings. The parameter combination with the best performance (*k* = 9 and *R* = 1.4) is highlighted with a larger cyan circle in the left panel. **B**: Adapter prediction performance on raw reads is shown with low complexity *k*-mers filtered out (left) and without the filtering step (right). The numbers in the panels indicate the number of correctly predicted adapters.

We first investigated the best parameter combination of *k* and *R* for DNApi in the *single* mode. For *k*-mer lengths from 8 to 13, *k* = 9 performed the best on both raw reads and quality trimmed reads as shown in Fig 2A. For *R* cutoffs from 1.1 to 2.0 with an increment of 0.1, *R* = 1.4 showed the best accuracy when combined with *k* = 9. The fraction of correctly estimated adapters (henceforth accuracy) reached 91.5% (493/539). Interestingly, the performance with raw reads showed better prediction accuracy compared to the ones with quality trimmed reads. We also tested the effect of low complexity filtering step and found that inclusion of the filtering step improves the prediction accuracy (Fig 2B; 476/539 without filtering). The accuracy of DNApi improved further to 92.0% when multiple *k*-*R* settings were considered (the *iterative* mode; 496/539). This shows the effectiveness of the iterative adapter search approach.

**Fig 3:**
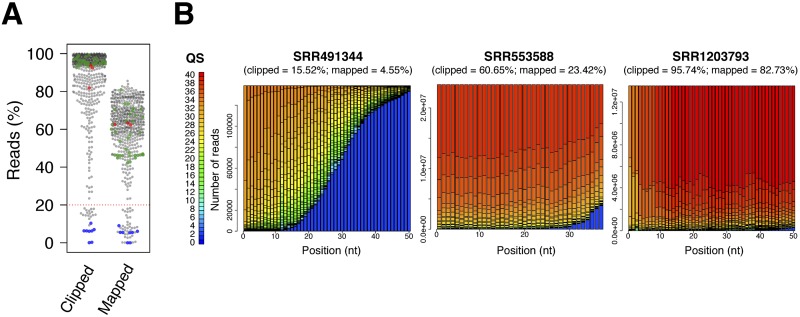
Quality assessment with the *exhaustive* mode in DNApi for the 539 libraries. **A**: Percentage of the reads for which the predicted 3´ adapter was clipped (“clipped”) and the reads mapped to the genome after adapter removal (“mapped”) are shown. Each dot represents a library. The 35 wrongly annotated libraries, 8 problematic libraries, and 3 libraries for which Minion failed to predict the adapters are highlighted in green, blue, and red respectively. The red line indicates the 20% cutoff. **B**: The distributions of quality scores in three libraries whose mapping rates were 4.6% (far below from the 20% cutoff of DNApi), 23.4% (slightly higher than the cutoff), and 82.7% (much higher than the cutoff) are shown. The percentage of clipped reads is also shown on top of each panel.

If a library is of high quality and the 3´ adapter is correctly predicted, a high percentage of reads should map to the reference genome. Fig 3A plots the fractions of reads clipped and mapped to the genome with the *exhaustive* mode in DNApi for the 539 libraries. The adapter prediction performance of the *exhaustive* mode was the same as the *iterative* mode (92.0%; 496/539). Among the 43 libraries for which the optimal adapters predicted by both *iterative* and *exhaustive* modes in DNApi disagreed with the metadata, 35 libraries were from one study [13]. When we used the 3´ adapters supplied by the authors, the genome mapping rates were ~1%. In sharp contrast, with our predicted adapters (listed in S3 Table) the mapping rates increased to ~80% (green dots in Fig 3A). We contacted the authors and it turned out our predicted adapters were correct and the metadata were in error. Two other libraries were control and upon immunoprecipitation of a human Argonaute protein [14], and most of the reads were ligation products of 5´ and 3´ adapters. The remaining 6 libraries came from the 40–100 nucleotide fraction of RNA purification [15]. While examining these libraries, we realized that few reads contained 3´ adapters because the RNA fragments were so long that the sequencing could not reach the 3´ adapters. These 8 problematic libraries are highlighted in blue dots in Fig 3A.

Correcting for the metadata of the 35 libraries, DNApi predicted 531 small RNA sequencing libraries with the correct adapter information (98.5%; 531/539).

To analyze small RNA libraries in a batch, it is also important to know whether the 3´ adapters have already been removed. The *exhaustive* mode in DNApi employs a user-defined read-mapping tool for mapping reads without adapter removal to investigate whether the adapter has been removed. Out of 1,708 small RNA libraries from human tissues in GEO as of November 2015, 192 libraries (S2 Table) were already processed according to their metadata. DNApi correctly determined that the adapters were already removed for all these libraries.

### Quality analysis on input small RNA libraries

Taking advantage of the large collection of the small RNA libraries, we investigated the mapping rates and the quality score distributions of the datasets. Fig 3A indicates that for 90.2% of the 539 libraries, more than 60% of the reads contained the predicted adapter, and for 87.4% of the libraries, more than 40% of the reads mapped to the genome after adapter removal. In addition to the 8 problematic libraries, only 37 libraries had mapping rates lower than 20%. Fig 3B shows a sharp contrast of quality score distributions between the libraries with the mapping rates over and under 20%, indicating that the libraries with lower than 20% mapping rates tend to have poor sequencing qualities.

### Comparison with Minion

**Fig 4:**
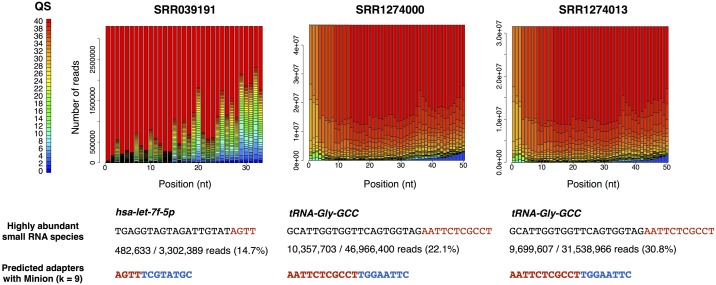
Quality score distribution and highly abundant small RNAs for the three libraries that Minion failed to predict the correct adapters. The distributions of quality scores for the three libraries that Minion wrongly predicted the 3´ adapter sequences are shown in three panels in the top row. Highly abundant small RNA species sequenced in the three libraries are shown in the middle row, and the wrongly predicted 3´ adapter sequences with Minion using 9-mer as the parameter are shown in the bottom row. The correct 3´ adapter sequences (8-nt long) and the parts of the highly abundant small RNA sequences are colored in blue and red respectively.

Using the 539 libraries with correct adapter information, we compared DNApi to the De Bruijn graph-based algorithm Minion in the Kraken package [16], which to the best of our knowledge is the only published tool for *de novo* adapter prediction. Like DNApi, Minion also did not predict the correct adaptor sequences for the aforementioned 8 problematic libraries. At its default *k*-mer setting (*k* = 12), Minion predicted the correct adapter sequences for 513 of the 539 libraries. Interestingly, Minion performs the best at *k* = 9, correctly identifying the adapter sequences for 528 libraries, which is the same performance as the *single* mode in DNApi with k = 9. However, Minion could not detect the correct adapters for the remaining 3 libraries even after combining the results of all different parameter settings (528/539). These 3 libraries contained highly abundant small RNAs (14.6%~30.8% of sequenced reads), and Minion wrongly predicted the 3´ portions of these small RNAs as part of the 3´ adapters (Fig 4). In contrast, the *iterative* and the *exhaustive* modes in DNApi identified the correct adapters (531/539), and the *exhaustive* mode judged the qualities of the 3 libraries as good (mapping rates = 62.31–63.36%; red dots in Fig 3A).

**Fig 5:**
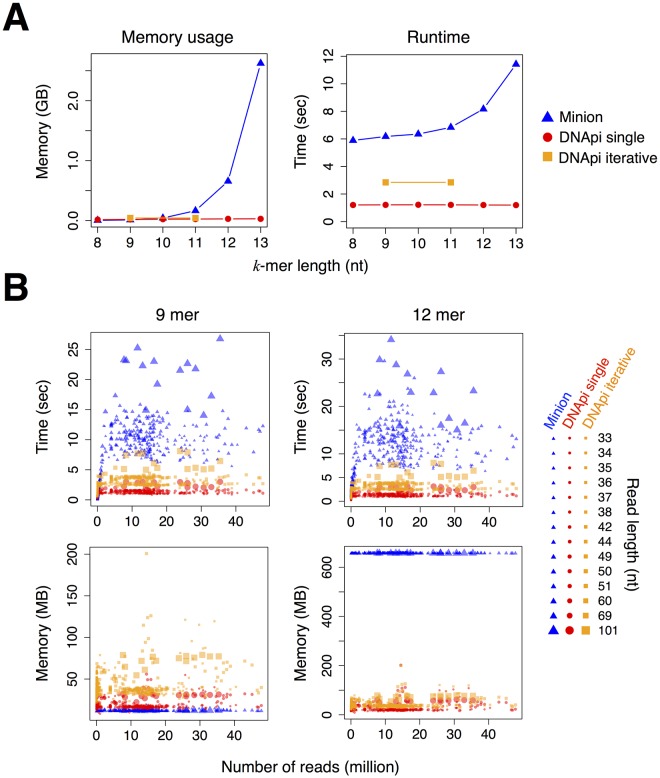
Adapter prediction performance of DNApi and Minion on the 539 small RNA libraries. **A**: Averaged memory usage and runtime of DNApi and Minion in different *k*-mer settings across all libraries are shown. The *iterative* mode in DNApi was run with *k* ∈ {9, 11}. **B**: Memory usage and runtime of DNApi and Minion in individual small RNA libraries are shown. *k* = 9 for the left panels and *k* = 12 for the right panels. The X-axis shows the sequencing depth of a library (total number of reads). The Y-axis of the upper panels is runtime in seconds and the Y-axis of the lower panels is memory usage in megabytes. Each library is shown as a symbol (circle and square for DNApi and triangle for Minion) with the size of the symbol indicating the read length of the library.

We also measured memory usage and runtime of Minion and DNApi (the *single* and *iterative* modes) on the 539 libraries. As averaged performance across all small RNA libraries, the *single* mode in DNApi shows fast runtime (~1.21 sec per library) and stably efficient memory usage (28 MB on average) in all *k*-mer settings as shown in Fig 5A. The *iterative* mode runs with slightly slower runtime (2.85 sec) and more memory (43 MB on average) than the *single* mode, however the difference is negligible. We further plotted the performance of DNApi and Minion for each individual small RNA library (Fig 5B). DNApi is faster than Minion, especially for libraries with longer reads. For small *k*, our algorithm uses slightly more memory than Minion, but for larger *k* (*k* = 12), our algorithm is much more memory efficient (mean = 26 MB in the *single* mode and 43 MB in the *iterative* mode) than Minion (mean = 657 MB).

### Limitations and future improvements

DNApi assumes that the most frequent *k*-mers in the sequencing reads originate from parts of the 3´ adapter sequence; thus, DNApi may not detect the correct adapter sequence when there is a large number of ligation products as we observed in two of the eight problematic libraries. To improve the prediction accuracy on those libraries, one may account for the patterns of how the 5´ and 3´ adapters are tandemly ligated. As for the remaining six problematic libraries in which few reads contain the 3´ adapter, the correct adapter sequence may be identified by mapping the reads to a reference genome and investigating the unmapped reads.

DNApi filters out *k*-mers composed of homopolymers. Another future development of DNApi is the detection of poly(A) or other low-complexity 3´ adapter sequences. Such homopolymeric adapters are confounded with unwanted low-complexity oligos such as the fragmented poly(A) tails of mRNAs. Some studies performed the polyadenylation reaction to extend the 3´ ends of small RNAs prior to sequencing [17,18]. In such cases, the 3´ adapters would be homopolymeric.

We also note two points regarding the effective use of DNApi. After removal of the correct adapter, the remaining cleansed reads should map to the reference genome at a high rate. For judging whether a library is already processed, the user would need a reference genome and use the *exhaustive* mode of DNApi. Another caveat is that DNApi does not perform de-multiplexing. Many powerful tools are available for de-multiplexing [19,20] and can be applied prior to running DNApi.

## Conclusion

We have developed DNApi that predicts the 3´ adapter sequence *de novo* and provides the user with cleansed small RNA sequences ready for down stream analysis. DNApi shows near-perfect accuracy on 539 publicly available small RNA libraries accompanied with 3´ adapter sequences in their metadata. DNApi also perfectly judged that 192 publicly available processed libraries were “ready-to-map” small RNA sequence reads. We hope that our tool DNApi will provide a convenient and accurate way to enable third-party meta-analysis of publicly available small RNA sequencing data. All the test data used for the evaluation (731 small RNA libraries from human samples) are listed in the Supporting Information tables, and readers can use the dataset for their biological analysis.

## Supporting Information

S1 Fig Sequencing quality and read length of 539 small RNA libraries. The fractions of remaining reads after quality trimming (Phred score cutoff ≥ 20) are plotted in the left panel, with the datasets grouped by the year of submission to the GEO. The fraction of remaining reads (i.e. the number of surviving reads after quality trimming divided by the total number of raw reads) reflects the quality of a small RNA library. Read lengths of the libraries are shown in the right panel. The numbers of the libraries in the years are: 2 in 2010, 245 in 2011, 56 in 2012, 151 in 2013, 75 in 2014, and 10 in 2015.
(TIFF)

S1 Table List of 539 publicly available small RNA libraries accompanied with 3´ adapters. (XLS)

S2 Table List of 192 publicly available processed libraries. (XLS)

S3 Table List of wrongly labeled adapters in the metadata and those correct adapters. (XLS)
