# Supplementary figures and images for: DNApi: A De Novo Adapter Prediction Algorithm for Small RNA Sequencing Data
Source: PLoS One. 2016 Oct 13;11(10):e0164228. doi: 10.1371/journal.pone.0164228 (PMC5063419; doi:10.1371/journal.pone.0164228)

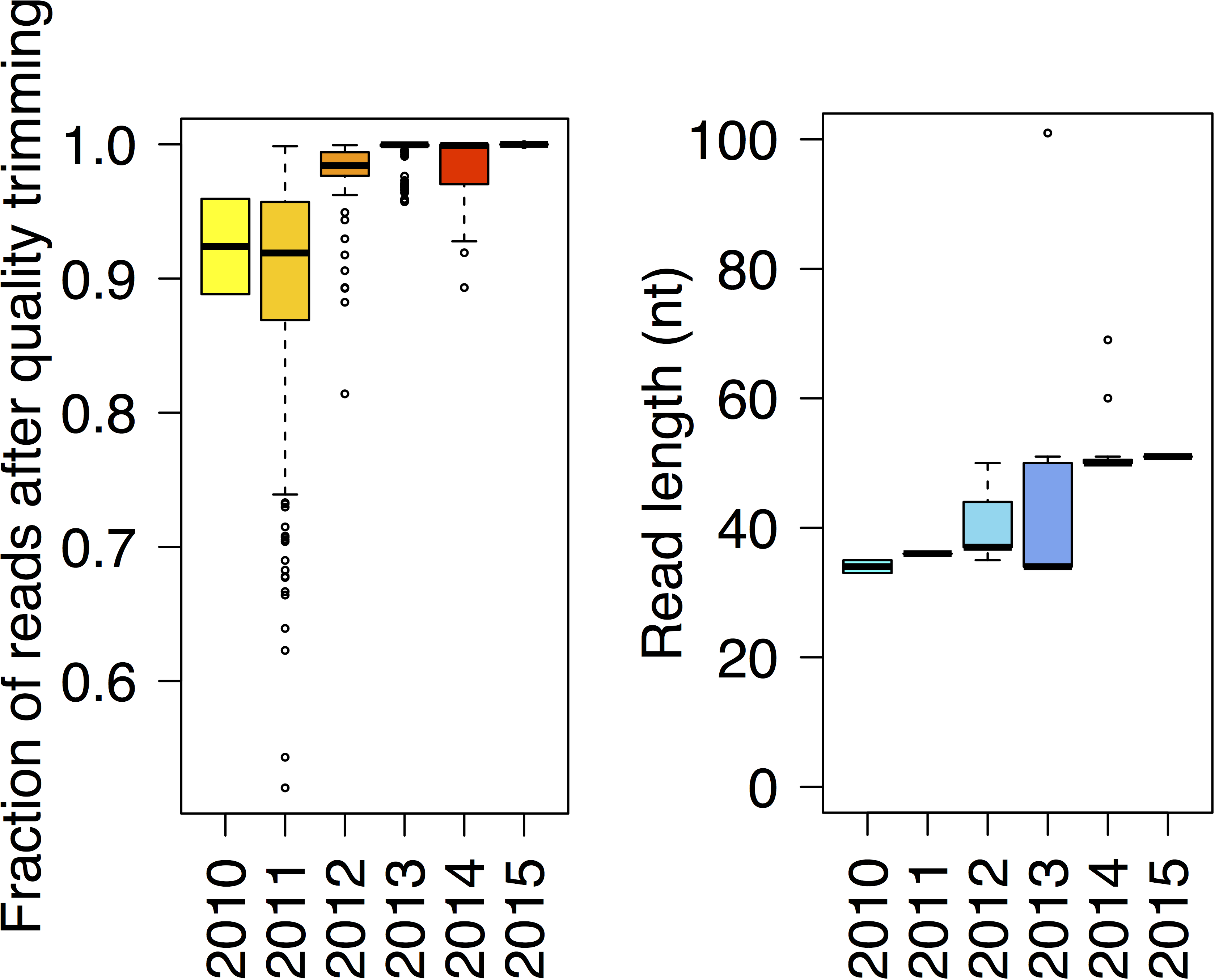

Supplement: S1 Fig — The fractions of remaining reads after quality trimming (Phred score cutoff ≥ 20) are plotted in the left panel, with the datasets grouped by the year of submission to the GEO. The fraction of remaining reads (i.e. the number of surviving reads after quality trimming divided by the total number of raw reads) reflects the quality of a small RNA library. Read lengths of the libraries are shown in the right panel. The numbers of the libraries in the years are: 2 in 2010, 245 in 2011, 56 in 2012, 151 in 2013, 75 in 2014, and 10 in 2015. (TIFF) [file pone.0164228.s001.tiff]
